# Revealing Variations in Perception of Mental States from Dynamic Facial Expressions: A Cautionary Note

**DOI:** 10.1371/journal.pone.0084395

**Published:** 2014-01-08

**Authors:** Elisa Back, Timothy R. Jordan

**Affiliations:** 1 Department of Psychology, Kingston University London, Kingston upon Thames, United Kingdom; 2 Department of Psychology, Zayed University, Dubai, United Arab Emirates; University of Leicester, United Kingdom

## Abstract

Although a great deal of research has been conducted on the recognition of basic facial emotions (e.g., anger, happiness, sadness), much less research has been carried out on the more subtle facial expressions of an individual's mental state (e.g., anxiety, disinterest, relief). Of particular concern is that these mental state expressions provide a crucial source of communication in everyday life but little is known about the accuracy with which natural dynamic facial expressions of mental states are identified and, in particular, the variability in mental state perception that is produced. Here we report the findings of two studies that investigated the accuracy and variability with which dynamic facial expressions of mental states were identified by participants. Both studies used stimuli carefully constructed using procedures adopted in previous research, and free-report (Study 1) and forced-choice (Study 2) measures of response accuracy and variability. The findings of both studies showed levels of response accuracy that were accompanied by substantial variation in the labels assigned by observers to each mental state. Thus, when mental states are identified from facial expressions in experiments, the identities attached to these expressions appear to vary considerably across individuals. This variability raises important issues for understanding the identification of mental states in everyday situations and for the use of responses in facial expression research.

## Introduction

The human face is an abundant source of visual information about a person's emotions, thoughts, and intentions, and the ability of observers to recognize this information is an invaluable skill for effective social communication. When researching these issues, numerous studies have investigated the processes underlying the recognition of basic emotions in faces (e.g., anger, happiness, sadness; [1]) with considerable success. However, relatively little research has investigated the ability to recognize the more subtle facial expressions of complex *mental states* (e.g., anxiety, disinterest, relief; [2]–[3]) that accompany everyday interactions.

The earliest investigations of the recognition of mental states used paintings and drawings of faces [4] or grey-scale photographs of the whole face and facial regions [2], [3]. These studies are seminal in mental state research, but static faces do not capture the nuances of facial movement made during the expression of mental states in everyday life. Indeed, several studies investigating face recognition [5], [6] and the recognition of basic emotions [7] suggest that dynamic information facilitates facial processing in general. Consequently, dynamic facial stimuli are important for developing a complete understanding of mental state recognition.

More recently [8], the role of dynamic information in mental state identification has been studied by comparing identification performance for dynamic facial expressions of various mental states with that of their matched static counterparts, displayed at the apex of each expression. The results showed a reliable advantage for dynamic facial stimuli, suggesting that the identification of mental states is sensitive to the dynamic properties of these expressions. Indeed, dynamic expressions of mental states have also been used to investigate clinical conditions such as autism [9], Williams syndrome [10], [11], and schizophrenia [12]. Thus, understanding the processing of naturalistic, dynamic facial expressions of mental states is likely to help provide a more complete picture of the processes involved in recognising mental states by clinical and non-clinical populations.

However, the effective use of dynamic facial stimuli in experiments designed to investigate the recognition of mental states requires even further consideration. Of particular importance is that mental state research using facial expressions has generally (and reasonably) determined recognition performance by considering the proportion of responses that match the identity of each portrayed mental state, and so adopts a binary approach to data in which responses are either correct or incorrect [2]–[4], [8], [13], [14]. But, in everyday life, the identification of mental states from dynamic facial expressions is unlikely to be so straightforward. In particular, due to the visual subtlety of this aspect of human communication, an observer may naturally produce a range of inferences about the facial information they see, and these inferences may also differ across observers. Consequently, when dynamic facial expressions of mental states are presented in experiments, the range of responses produced by each stimulus is likely to provide important indications about the processing that leads to the inference of mental state identity, and the complexity of this recognition process may not be adequately represented solely by the number of correct responses made in experiments.

The issue of variations in responses to facial stimuli is highlighted in a recent study using a broad range of emotional and conversational facial expressions [15]. A set of dynamic facial stimuli was carefully constructed and presented to 10 participants who then assigned a verbal label to each stimulus. These labels were then rated as valid or invalid by a separate group of three judges. However, despite the care with which stimuli were constructed, these procedures revealed that only 60% of all responses to dynamic facial stimuli were judged as valid. Furthermore, within the range of valid responses, the labels used to identify facial expressions varied considerably, and this variability was observed over a wide range of facial expressions.

The variation in responses produced by facial expressions of mental states has not been addressed in previous research, despite the influence that such variability is likely to have on identification performance. This is of particular concern because previous studies of mental state recognition tend not to state clearly how their facial expression stimuli have been validated and, when validation measures have been used, they often do not appear to be particularly rigorous. For example, in one study [4], a number of facial expressions were presented, each representing a particular mental state and one of the authors suggested a word to describe each expression for the experiment. If three other authors were in agreement, this word was selected as the label for that stimulus. In another study [2], an actor posed facial expressions, a word was chosen to describe the mental state portrayed by each expression by four independent judges, and a word was selected as the label if it received unanimous agreement. These validation methods may be problematic because gathering decisions from only a small number of judges is likely to inflate the apparent suitability of a label for a stimulus. The stimuli constructed for a computer software program [16] were validated by ten judges, and facial expressions were included if eight or more judges agreed on the label [17]. No further details were provided on how they reached agreement and the absence of reporting how mental state stimuli are validated is often the case with research in this area (e.g., [13], [14]). Moreover, many studies have been limited to presenting the same forced-choice options to validate the identity of each facial expression (i.e., presenting the same labels for each face) and it has been acknowledged that this can inflate levels of agreement [18]. Against this background, the aim of the present study was to investigate the variability in identification of mental states produced by natural dynamic facial expressions of a range of mental states used typically in mental state research. The variability in the responses produced by these stimuli was assessed in two ways: by a free-report procedure in Study 1, and by a forced-choice procedure in Study 2.

## Methods

### Ethics Statement

The studies presented here involved human volunteers and these studies were approved by the School of Psychology Research Ethics Committee at the University of Nottingham. Informed written consent was obtained from all participants and the British Psychology Society's ethical guidelines were followed closely.

### Preparation of stimuli and filming procedure

A set of 25 mental states was compiled from previous research [2], [3], [4]. These mental states were: admiring, amazed, amused, anguish, annoyed, anxious, ashamed, confident, confused, disinterest, distrustful, embarrassed, excited, flirtatious, guilty, jealous, pain, panicked, preoccupied, quizzical, relieved, scheming, stern, thoughtful, and unfriendly. A list of these mental states, with definitions, was given to an actor (female, 22 years old) together with an example situation for each mental state to aid the actor's understanding; for example, “think of a time when you were amused by a funny joke that someone told you”. This procedure was adapted from previous studies [2], [15] and was designed to help the actor recall past experiences to bring about the state of mind required. This helped produce more realistic facial expressions and was based on the Stanislawski technique [19] where actors retrieve past experiences that evoke the emotions or mental states they are asked to enact. Similar to the procedure of a previous study [15], to avoid stereotypical expressions and to enhance the lifelike nature of the expressions that were made, the actor used to portray each mental state did not have formal acting training.

Each mental state was read aloud to the actor one at a time and the face of the actor was recorded while she enacted each mental state. The face was fully illuminated and recorded life-sized and in natural colour while fixating a digital video camera (Sony DSR200-AP), with the head stationary against a uniform white background so that only the face was visible. The actor showed her face in neutral repose for a few seconds before producing each facial expression and returned to the neutral repose after each expression. Several takes were recorded for each of the 25 expressions.

### Editing stimuli

Raw captured footage was imported into post-production editing software [20]. Individual clips were created for each facial expression and were edited to show the face in neutral repose for one second before the onset of the facial expression and in neutral repose for one second after the offset. The duration of the apex in each of the dynamic facial expressions averaged approximately five seconds. Clips were then imported into a software program [21] where they were exported as quick-time movies and made into quality-enhanced images that ran in real-time (25 frames per second).

To maintain close similarity to the selection procedures used in previous studies (e.g., [2]), four expert members of our research group selected the best example of each mental state. However, although the procedure we used to select clips was deliberately typical of that used in previous studies, the identification of each of the 25 mental state stimuli was assessed in two separate studies.

In Study 1, a free-report procedure was used in which participants were required to generate an appropriate mental state label for each facial expression. This procedure was adopted to provide an unrestricted indication of the variability in responses that participants make when required to identify mental states in experiments. In Study 2, a forced-choice procedure was used in which a set of responses was assembled for each expression using the data obtained in Study 1. In particular, participants were required to choose a label from a set of five words that included the expressed mental state and the four most common alternative responses for the same stimulus from Study 1. In this way, participants were required to select a label from a closed set of valid responses, and this provided an important measure of the influence of free-report and forced-choice on response variability in mental state research.

## Study 1

### Method

#### Participants

Sixteen participants (undergraduates and postgraduates) from the University of Nottingham took part. All had English as their first language and normal or corrected to normal vision.

#### Stimuli

The 25 QuickTime movie clips of facial expressions were presented on a high-definition 22 inch flat screen monitor using PsyScope [22]. Each face subtended approximately 9° vertically and 6° horizontally at the viewing distance of 1 m.

#### Design and Procedure

Each participant viewed all clips four times, once in each of four separate blocks, each block consisting of the 25 clips. Facial expressions within each block were presented in a different random order for each participant. After viewing each clip, participants were asked to report the word that most accurately described what the person showing the expression was thinking or feeling.

### Results and Discussion

Percentage totals and cumulative frequency totals of all participants' free-report responses were calculated for each facial expression. The four words that were the most commonly reported for each facial expression and the number of participants that reported each word for each stimulus are shown in Table S1. Generally, there appeared to be little agreement amongst participants over a single freely-reported word to describe a particular facial expression. We shall return to these findings in the General Discussion.

As a range of responses were generated from the free-report procedures, the next development was to investigate, for each stimulus, participants' choices from a set of words that included the enacted mental state and the most common four free-report responses from Study 1. This procedure was chosen to provide participants with the opportunity of selecting a label that they may not generate themselves but that they still thought was the most appropriate. For example, in Study 1, labels for basic emotions, such as *happy* and *sad*, were generated frequently as responses and these may have been chosen rather than mental states, such as *excited* and *disapprovin*g, because mental state labels are less common than basic emotion labels in everyday language. It was therefore important to determine whether participants would select a mental state identity when given the choice.

## Study 2

### Method

#### Participants

A different sample of sixteen participants, from the same population as Study 1, took part in the experiment. All had English as their first language and normal or corrected to normal vision.

#### Procedure

The study used the same stimuli, design and procedure as before except that each clip was now followed by the presentation of five words on the screen, corresponding to the enacted mental state and the four most common responses for that mental state from Study 1 (see Table S2). Participants were asked to choose the word that most accurately described what the person showing the expression was thinking or feeling by selecting that option.

### Results

Percentage totals and cumulative frequency totals of all participants' responses were calculated for each facial expression and the numbers of participants (maximum 16) reporting each word at least once appear in Table S2. For fourteen mental states, the two most common responses were chosen over 60% of the time. Table 1 shows the percentage of times that each of these mental states (most common response) and the closest alternative (second most common response) were chosen by participants.

**Table 1:** Percentage rates for selected mental states and alternatives (the second most commonly chosen word).

Percentage Threshold	Enacted mental state	Mental state (and alternative)	Percentage
100%	None	None	0%
90%	Thinking	Thinking(guilt)	94%
80%	Amazed	Surprised(shocked)	86%
	Anxious	Anxious(nervous)	88%
	Flirtatious	Flirtatious(playful)	81%
	Confused	Bemused(disbelief)	81%
70%	Admiring	Considering(approving)	78%
	Disinterest	Disinterest(disapproving)	78%
	Scheming	Suspicious(unsure)	75%
	Unfriendly	Disapproving(disgusted)	72%
60%	Stern	Stern(unsure)	69%
	Relieved	Relieved(impatient)	66%
	Confident	Smug(unsure)	66%
	Quizzical	Doubtful(uncertainty)	64%
	Distrustful	Distrustful(doubtful)	61%

## General Discussion

The findings of this research show that identification of naturally-dynamic facial expressions of mental states is much more variable than indicated by previous research. Indeed, observers in both studies made a wide range of responses to each stimulus, suggesting that attempting to define mental state recognition performance in experiments merely by using the proportion of responses that match the assigned identity of each mental state is only one way of assessing mental state recognition and is unlikely to provide the complete picture. Moreover, our findings show that the range of responses produced cannot be accounted for by close, semantic alternatives, and can represent very different mental states. For example, the portrayed mental state “thinking” elicited numerous free-report responses of “guilt” and “hopeful”, and the portrayed mental state “scheming” elicited “annoyed” and “unsure”. In addition, providing explicit alternatives in the forced-choice version of the experiment did not remove response variability, indicating that the wide range of responses observed in this research is not restricted to just one method of assessment. A further interesting finding is that individual raters would sometimes attribute different words each time the same stimulus was presented and this may suggest that judging mental states can be influenced by the preceding stimuli and that raters generally may vary in their use of labels for mental state stimuli within an experiment [23].

The observed variability in responses provides important indications about the processes involved in recognizing mental states. In particular, the findings suggest that different individuals can perceive the facial expression of a mental state in very different ways, and so may attribute a range of different states of mind to the same facial expression. The subtlety of expression in mental states may play a part in this variability, and in everyday life other cues may be critical to help observers determine the actual mental state of an individual. For example, body pose, hand gestures, environmental and situational cues, and conversational context can all provide additional valuable information. Clearly, from our findings, facial expressions alone, even the natural dynamic images used in our study, are not capable of providing unequivocal identification of mental states, suggesting that a full understanding of the role of facial expressions in mental state recognition requires a more holistic approach. But, of course, mistakes in identifying the mental states of individuals do get made in everyday life, and the variation in responses we obtained throws new light on just how challenging the accurate recognition of mental states from facial information actually is. Future research would benefit from including a range of actors expressing mental states and additional ratings of valence and arousal could provide a more comprehensive understanding of how mental states are perceived and subsequently processed.

The findings of this research are all the more relevant because the mental state stimuli used in both studies were naturally dynamic and enacted using well-established protocols (e.g., [3], [15]), and were validated in Study 1 and Study 2 using procedures more stringent than those typical in mental state research. In particular, both procedures were an improvement on many previous studies that have relied on the judgements of only a small number of judges to validate the identity of each facial stimulus [2]–[4] and the findings highlight the dangers that researchers investigating perception of mental states from facial expressions are likely to encounter when relying on a single term to describe a facial expression. Indeed, the appropriateness of a particular label assigned by researchers to describe a facial expression may vary considerably across participants, and may be affected greatly by individual vocabularies, which may not be homogeneous even at similar levels of development. Consequently, attention to this variation may be especially crucial for investigating mental state recognition in specific groups of participants, and particularly for typically developing children, children with autism spectrum conditions [9], and individuals with Williams syndrome [10], [11], where the mental state terms and respective foils used in experiments should be validated appropriately for these groups.

Attributing the correct label to facial expressions of mental states is a difficult process and some studies [24], [25] highlight the importance that language plays in providing an “internal context” when recognising emotions, which helps humans determine how emotions are perceived. Therefore, attributing a verbal label to a facial expression is an important aspect of studying perception of mental states and offers more than procedures that may rely just on the ability to sort and match facial expressions from perceptual characteristics alone.

Indeed, our findings suggest that caution is required when interpreting the results of previous studies of mental state recognition that have not undertaken rigorous validation methods and have overlooked the variation that exists amongst individuals describing particular facial expressions, especially when using very few judges to determine the labels that were used. Future research would do well to ensure that the perceived identities of facial expressions are more rigorously validated using a greater number of judges, and a combination of free-report and forced-choice methodologies.

## Supporting Information

Table S1 **Most common free-report responses for each facial expression.**
(DOCX)

Table S2 **The number of times participants chose a particular word (mental state) to describe each facial expression.**
(DOCX)
