# Supplementary material for: Revealing Variations in Perception of Mental States from Dynamic Facial Expressions: A Cautionary Note
Source: PLoS One. 2014 Jan 8;9(1):e84395. doi: 10.1371/journal.pone.0084395 (PMC3885558; doi:10.1371/journal.pone.0084395)
Supplement: Table S1 — Most common free-report responses for each facial expression. (DOCX) [file pone.0084395.s001.docx]

| Mental state | Response | Frequency | Number of participants reporting each word at least once | Percentage | Cumulative  frequency percentage |
| --- | --- | --- | --- | --- | --- |
|  | Considering | 5 | 3 | 7.81 | 7.81 |
| Admiring | Approving | 4 | 3 | 6.25 | 14.06 |
|  | Ok | 4 | 2 | 6.25 | 20.31 |
|  | Fed-up | 3 | 1 | 4.69 | 25 |
|  | Surprised | 45 | 15 | 70.31 | 70.31 |
| Amazed | Shocked | 4 | 4 | 6.25 | 76.56 |
|  | Happy | 3 | 2 | 4.69 | 81.25 |
|  | Pleased | 2 | 2 | 3.13 | 84.38 |
|  | Happy | 20 | 9 | 31.25 | 31.25 |
| Amused | Amused | 11 | 5 | 17.19 | 48.44 |
|  | Pleased | 9 | 4 | 14.06 | 62.5 |
|  | Embarrassed | 3 | 1 | 4.69 | 67.19 |
|  | Pain | 11 | 3 | 17.19 | 17.19 |
| Anguish | Scared | 9 | 5 | 14.06 | 31.25 |
|  | Anxious | 5 | 3 | 7.81 | 39.06 |
|  | Worried | 4 | 2 | 6.25 | 45.31 |
|  | Annoyed | 17 | 10 | 26.56 | 26.56 |
| Annoyed | Confused | 8 | 5 | 12.5 | 39.06 |
|  | Angry | 4 | 3 | 6.25 | 45.31 |
|  | Doubtful | 4 | 3 | 6.25 | 51.56 |
|  | Worried | 8 | 4 | 12.5 | 12.5 |
| Anxious | Nervous | 8 | 4 | 12.5 | 25 |
|  | Doubtful | 4 | 4 | 6.25 | 31.25 |
|  | Sympathetic | 4 | 4 | 6.25 | 37.5 |
|  | Sad | 9 | 7 | 14.06 | 14.06 |
| Ashamed | Upset | 9 | 5 | 14.06 | 28.13 |
|  | Unsure | 5 | 3 | 7.81 | 35.94 |
|  | Disappointed | 5 | 1 | 7.81 | 43.75 |
|  | Annoyed | 9 | 6 | 14.06 | 14.06 |
| Confident | Smug | 9 | 6 | 14.06 | 28.13 |
|  | Pleased | 6 | 3 | 9.38 | 37.5 |
|  | Unsure | 4 | 1 | 6.25 | 43.75 |
|  | Surprised | 7 | 7 | 9.38 | 9.38 |
| Confused | Amused | 5 | 4 | 7.81 | 17.19 |
|  | Bemused | 5 | 2 | 7.81 | 25 |
|  | Confused | 5 | 3 | 7.81 | 32.81 |
|  | Resigned | 8 | 4 | 12.5 | 12.5 |
| Disinterest | Bored | 7 | 4 | 10.94 | 23.44 |
|  | Disappointed | 5 | 4 | 9.38 | 32.81 |
|  | Unsure | 5 | 5 | 7.81 | 40.63 |
|  | Annoyed | 13 | 6 | 20.31 | 20.31 |
| Distrustful | Confused | 5 | 3 | 7.81 | 28.13 |
|  | Unsure | 5 | 5 | 7.81 | 35.94 |
|  | Doubtful | 5 | 4 | 7.81 | 43.75 |
|  | Embarrassed | 8 | 6 | 12.5 | 12.5 |
| Embarrassed | Nervous | 7 | 4 | 10.94 | 23.44 |
|  | Pain | 7 | 3 | 10.94 | 34.38 |
|  | Worried | 7 | 5 | 10.94 | 45.31 |
|  | Happy | 39 | 13 | 60.94 | 60.94 |
| Excited | Cheeky | 4 | 1 | 6.25 | 67.19 |
|  | Excited | 4 | 1 | 6.25 | 73.44 |
|  | Pleased | 3 | 2 | 4.69 | 78.13 |
|  | Flirty | 8 | 4 | 12.5 | 12.5 |
| Flirtatious | Smug | 5 | 3 | 7.81 | 20.31 |
|  | Flirtatious | 5 | 3 | 7.81 | 28.13 |
|  | Cocky | 4 | 2 | 6.25 | 34.38 |
|  | Worried | 13 | 5 | 20.31 | 20.31 |
| Guilty | Sad | 7 | 5 | 10.93 | 31.25 |
|  | Disappointed | 4 | 3 | 6.25 | 37.5 |
|  | Scared | 3 | 3 | 4.69 | 42.19 |
|  | Annoyed | 10 | 6 | 15.63 | 15.63 |
| Jealousy | Thoughtful | 10 | 7 | 15.63 | 15.63 |
|  | Angry | 8 | 5 | 12.5 | 43.75 |
|  | Cross | 3 | 1 | 4.69 | 48.44 |
|  | Pain | 8 | 3 | 12.5 | 12.5 |
| Pain | Cringing | 6 | 4 | 9.38 | 21.88 |
|  | Ouch | 6 | 3 | 9.38 | 31.25 |
|  | Scared | 4 | 3 | 6.25 | 37.5 |
|  | Scared | 18 | 8 | 28.13 | 28.13 |
| Panicked | Shocked | 11 | 5 | 17.19 | 45.31 |
|  | Frightened | 11 | 5 | 17.19 | 62.5 |
|  | Fear | 7 | 2 | 10.94 | 73.44 |
|  | Disappointed | 11 | 5 | 17.19 | 17.19 |
| Preoccupied | Sad | 10 | 6 | 15.63 | 32.81 |
|  | Unsure | 5 | 5 | 7.81 | 40.63 |
|  | Unhappy | 3 | 3 | 4.69 | 45.31 |
|  | Confused | 11 | 7 | 17.19 | 17.19 |
| Quizzical | Annoyed | 8 | 6 | 12.5 | 29.69 |
|  | Doubtful | 7 | 3 | 10.94 | 40.63 |
|  | Uncertainty | 5 | 3 | 7.81 | 48.44 |
|  | Relieved | 39 | 12 | 60.94 | 60.94 |
| Relieved | Impatient | 5 | 3 | 7.81 | 68.75 |
|  | Annoyed | 4 | 1 | 6.25 | 75 |
|  | Fed-up | 4 | 1 | 6.25 | 81.25 |
|  | Unsure | 10 | 7 | 15.63 | 15.63 |
| Scheming | Annoyed | 6 | 3 | 9.38 | 25 |
|  | Suspicious | 4 | 2 | 6.25 | 31.25 |
|  | Confused | 3 | 2 | 4.69 | 35.94 |
|  | Unsure | 13 | 9 | 20.31 | 20.31 |
| Stern | Annoyed | 10 | 3 | 15.63 | 35.94 |
|  | Confused | 7 | 6 | 10.94 | 46.88 |
|  | Disapproving | 4 | 3 | 6.25 | 53.13 |
|  | Thinking | 11 | 6 | 17.19 | 17.19 |
| Thinking | Thoughtful | 7 | 3 | 10.94 | 28.13 |
|  | Guilt | 7 | 2 | 10.94 | 39.07 |
|  | Pensive | 5 | 3 | 7.81 | 46.88 |
|  | Confused | 7 | 6 | 10.94 | 10.94 |
| Unfriendly | Annoyed | 6 | 3 | 9.38 | 20.32 |
|  | Disapproving | 4 | 3 | 6.25 | 26.57 |
|  | Disgusted | 4 | 4 | 6.25 | 32.82 |
