# Supplementary material for: Revealing Variations in Perception of Mental States from Dynamic Facial Expressions: A Cautionary Note
Source: PLoS One. 2014 Jan 8;9(1):e84395. doi: 10.1371/journal.pone.0084395 (PMC3885558; doi:10.1371/journal.pone.0084395)
Supplement: Table S2 — The number of times participants chose a particular word (mental state) to describe each facial expression. (DOCX) [file pone.0084395.s002.docx]

| Mental state | Response | Frequency | Number of participants reporting word at least once | Percentage | Cumulative frequency percentage |
| --- | --- | --- | --- | --- | --- |
|  | Considering | 37 | 12 | 57.81 | 57.81 |
|  | Approving | 13 | 6 | 20.31 | 78.12 |
| Admiring | Okay | 8 | 4 | 12.5 | 90.62 |
|  | Admiring | 3 | 2 | 4.69 | 95.28 |
|  | Fed-up | 3 | 3 | 4.69 | 100 |
|  | Surprised | 50 | 16 | 78.13 | 78.13 |
|  | Shocked | 5 | 2 | 7.81 | 85.94 |
| Amazed | Amazed | 4 | 4 | 6.25 | 92.19 |
|  | Pleased | 3 | 3 | 4.69 | 96.88 |
|  | Happy | 2 | 2 | 3.13 | 100 |
|  | Pleased | 21 | 12 | 32.81 | 32.81 |
|  | Amused | 15 | 8 | 23.44 | 56.25 |
| Amused | Embarrassed | 14 | 6 | 21.88 | 78.13 |
|  | Happy | 13 | 7 | 20.31 | 98.44 |
|  | Excited | 1 | 1 | 1.56 | 100 |
|  | Anxious | 31 | 15 | 48.44 | 48.44 |
|  | Worried | 17 | 10 | 26.6 | 75.04 |
| Anguish | Pain | 8 | 5 | 12.5 | 87.54 |
|  | Anguish | 5 | 3 | 7.81 | 95.3 |
|  | Scared | 3 | 3 | 4.69 | 100 |
|  | Annoyed | 24 | 12 | 37.5 | 37.5 |
|  | Doubtful | 21 | 11 | 32.81 | 70.31 |
| Annoyed | Puzzled | 12 | 7 | 18.75 | 89.06 |
|  | Confused | 5 | 4 | 7.81 | 96.87 |
|  | Angry | 2 | 2 | 3.13 | 100 |
|  | Anxious | 21 | 12 | 32.81 | 32.81 |
|  | Worried | 21 | 12 | 32.81 | 65.62 |
| Anxious | Nervous | 14 | 8 | 21.88 | 87.5 |
|  | Doubtful | 6 | 5 | 9.38 | 96.88 |
|  | Sympathetic | 2 | 1 | 3.13 | 100 |
|  | Sad | 24 | 10 | 37.5 | 37.5 |
|  | Unsure | 15 | 6 | 23.44 | 60.94 |
| Ashamed | Upset | 14 | 7 | 21.88 | 82.82 |
|  | Ashamed | 8 | 5 | 12.5 | 95.32 |
|  | Disappointed | 3 | 3 | 4.69 | 100 |
|  | Smug | 30 | 10 | 46.88 | 46.88 |
|  | Unsure | 12 | 8 | 18.75 | 65.63 |
| Confident | Confident | 11 | 6 | 17.19 | 82.82 |
|  | Annoyed | 9 | 4 | 14.06 | 96.88 |
|  | Pleased | 2 | 2 | 3.13 | 100 |
|  | Bemused | 37 | 12 | 57.81 | 57.81 |
|  | Disbelief | 15 | 7 | 23.44 | 81.25 |
| Confused | Amused | 7 | 5 | 10.94 | 92.19 |
|  | Confused | 4 | 2 | 6.25 | 98.44 |
|  | Surprised | 1 | 1 | 1.56 | 100 |
|  | Disinterest | 37 | 12 | 57.81 | 57.81 |
|  | Disapproving | 13 | 8 | 20.31 | 78.12 |
| Disinterest | Bored | 11 | 4 | 17.19 | 95.31 |
|  | Disappointed | 2 | 2 | 3.13 | 98.44 |
|  | Annoyed | 1 | 1 | 1.56 | 100 |
|  | Distrustful | 22 | 10 | 34.38 | 34.38 |
|  | Doubtful | 17 | 9 | 26.56 | 60.94 |
| Distrustful | Annoyed | 13 | 9 | 20.31 | 81.25 |
|  | Unsure | 10 | 4 | 15.63 | 96.88 |
|  | Confused | 2 | 2 | 3.13 | 100 |
|  | Anxious | 26 | 12 | 40.63 | 40.63 |
|  | Embarrassed | 22 | 9 | 34.38 | 75.01 |
| Embarrassed | Worried | 8 | 6 | 12.5 | 87.51 |
|  | Nervous | 7 | 5 | 10.94 | 98.45 |
|  | Pain | 1 | 1 | 1.56 | 100 |
|  | Happy | 38 | 13 | 59.38 | 59.38 |
|  | Pleased | 13 | 8 | 20.31 | 79.69 |
| Excited | Excited | 8 | 4 | 12.5 | 92.19 |
|  | Joy | 3 | 3 | 4.69 | 96.88 |
|  | Cheeky | 2 | 2 | 3.13 | 100 |
|  | Flirtatious | 36 | 12 | 56.25 | 56.25 |
|  | Playful | 16 | 6 | 25 | 81.25 |
| Flirtatious | Flirty | 6 | 4 | 9.38 | 90.63 |
|  | Smug | 5 | 4 | 7.81 | 98.44 |
|  | Cocky | 1 | 1 | 1.56 | 100 |
|  | Worried | 32 | 12 | 50 | 50 |
|  | Sad | 16 | 9 | 25 | 75 |
| Guilty | Guilty | 8 | 5 | 12.5 | 87.5 |
|  | Disappointed | 8 | 4 | 12.5 | 100 |
|  | Scared | 0 | 0 | 0 | 100 |
|  | Annoyed | 25 | 11 | 39.06 | 39.06 |
|  | Thoughtful | 13 | 6 | 20.31 | 59.37 |
| Jealousy | Cross | 12 | 7 | 18.75 | 78.12 |
|  | Angry | 9 | 5 | 14.06 | 92.18 |
|  | Jealousy | 5 | 2 | 7.81 | 100 |
|  | Cringing | 42 | 12 | 66.63 | 65.63 |
|  | Ouch | 13 | 1 | 20.31 | 85.94 |
| Pain | Pain | 8 | 5 | 12.5 | 98.44 |
|  | Anxious | 1 | 5 | 1.56 | 100 |
|  | Scared | 0 | 0 | 0 | 100 |
|  | Frightened | 23 | 11 | 35.94 | 35.94 |
|  | Scared | 16 | 7 | 25 | 60.94 |
| Panicked | Shocked | 10 | 6 | 15.63 | 76.57 |
|  | Panicked | 8 | 4 | 12.5 | 89.07 |
|  | Fear | 7 | 3 | 10.94 | 100 |
|  | Reflective | 21 | 11 | 32.81 | 32.81 |
|  | Disappointed | 19 | 10 | 29.69 | 62.5 |
| Preoccupied | Thoughtful | 15 | 8 | 23.44 | 85.94 |
|  | Preoccupied | 7 | 7 | 10.94 | 96.88 |
|  | Sad | 2 | 2 | 3.13 | 100 |
|  | Doubtful | 29 | 13 | 45.31 | 45.31 |
|  | Uncertainty | 12 | 8 | 18.75 | 64.06 |
| Quizzical | Quizzical | 10 | 7 | 15.63 | 79.69 |
|  | Annoyed | 8 | 4 | 12.5 | 92.19 |
|  | Confused | 5 | 4 | 7.81 | 100 |
|  | Relieved | 25 | 9 | 39.06 | 39.06 |
|  | Impatient | 17 | 9 | 26.56 | 65.62 |
| Relieved | Bored | 11 | 6 | 17.19 | 82.81 |
|  | Fed-up | 9 | 5 | 14.06 | 96.87 |
|  | Annoyed | 2 | 2 | 3.13 | 100 |
|  | Suspicious | 28 | 16 | 43.75 | 43.75 |
|  | Unsure | 20 | 11 | 31.25 | 75 |
| Scheming | Scheming | 9 | 7 | 14.06 | 89.06 |
|  | Annoyed | 6 | 4 | 9.38 | 98.44 |
|  | Confused | 1 | 1 | 1.56 | 100 |
|  | Disapproving | 28 | 12 | 43.75 | 43.75 |
|  | Unsure | 16 | 10 | 25 | 68.75 |
| Stern | Stern | 9 | 6 | 14.06 | 82.81 |
|  | Annoyed | 8 | 5 | 12.5 | 95.31 |
|  | Confused | 3 | 3 | 4.69 | 100 |
|  | Guilt | 19 | 7 | 29.69 | 29.69 |
|  | Pensive | 15 | 6 | 23.44 | 53.13 |
| Thinking | Thoughtful | 14 | 6 | 21.88 | 75.01 |
|  | Thinking | 12 | 6 | 18.75 | 93.76 |
|  | Hopeful | 4 | 4 | 6.25 | 100 |
|  | Disapproving | 35 | 14 | 54.69 | 54.69 |
|  | Disgusted | 11 | 6 | 17.19 | 71.88 |
| Unfriendly | Unfriendly | 9 | 4 | 14.06 | 85.94 |
|  | Annoyed | 5 | 4 | 7.81 | 93.75 |
|  | Confused | 4 | 3 | 6.25 | 100 |
